# Cardiometabolic disease risk and HIV status in rural South Africa: establishing a baseline

**DOI:** 10.1186/s12889-015-1467-1

**Published:** 2015-02-12

**Authors:** Samuel J Clark, F Xavier Gómez-Olivé, Brian Houle, Margaret Thorogood, Kerstin Klipstein-Grobusch, Nicole Angotti, Chodziwadziwa Kabudula, Jill Williams, Jane Menken, Stephen Tollman

**Affiliations:** MRC/Wits Rural Public Health and Health Transitions Research Unit, School of Public Health, Faculty of Health Sciences, University of the Witwatersrand, Johannesburg, South Africa; Institute of Behavioral Science, University of Colorado Boulder, Boulder, USA; School of Public Health, Faculty of Health Sciences, University of the Witwatersrand, Johannesburg, South Africa; Julius Global Health, Julius Center for Health Sciences and Primary Care, University Medical Center Utrecht, Utrecht University, Utrecht, Netherlands; Centre for Global Health Research, Umeå University, Umeå, Sweden; INDEPTH Network, Accra, Ghana; Department of Sociology, University of Washington, Seattle, USA; Division of Health Sciences, University of Warwick Medical School, Coventry, UK; Australian Demographic and Social Research Institute, Australian National University, Canberra, Australia; Department of Sociology and Center on Health, Risk, and Society, American University, Washington, DC USA

**Keywords:** South Africa, Rural, Cardiometabolic risk, HIV/AIDS

## Abstract

**Background:**

To inform health care and training, resource and research priorities, it is essential to establish how non-communicable disease risk factors vary by HIV-status in high HIV burden areas; and whether long-term anti-retroviral therapy (ART) plays a modifying role.

**Methods:**

As part of a cohort initiation, we conducted a baseline HIV/cardiometabolic risk factor survey in 2010–2011 using an age-sex stratified random sample of ages 15+ in rural South Africa. We modelled cardiometabolic risk factors and their associations by HIV-status and self-reported ART status for ages 18+ using sex-stratified logistic regression models.

**Results:**

Age-standardised HIV prevalence in women was 26% (95% CI 24-28%) and 19% (95% CI 17–21) in men. People with untreated HIV were less likely to have a high waist circumference in both women (OR 0.67; 95% CI 0.52-0.86) and men (OR 0.42; 95% CI 0.22-0.82). Untreated women were more likely to have low HDL and LDL, and treated women high triglycerides. Cardiometabolic risk factors increased with age except low HDL. The prevalence of hypertension was high (40% in women; 30% in men).

**Conclusions:**

Sub-Saharan Africa is facing intersecting epidemics of HIV and hypertension. In this setting, around half the adult population require long-term care for at least one of HIV, hypertension or diabetes. Together with the adverse effects that HIV and its treatment have on lipids, this may have serious implications for the South African health care system. Monitoring of the interaction of HIV, ART use, and cardiometabolic disease is needed at both individual and population levels.

**Electronic supplementary material:**

The online version of this article (doi:10.1186/s12889-015-1467-1) contains supplementary material, which is available to authorized users.

## Background

Reflecting a complex health transition, the population of South Africa is ageing as widespread lifestyle changes occur and antiretroviral therapy (ART) for HIV/AIDS becomes more readily available. Thus a co-morbid burden of cardiometabolic disease and HIV/AIDS is emerging. The South African population carries a rising prevalence of cardiometabolic diseases and their risk factors [1-6], including poor communities in rural South African settings [7]. Mortality from cardiometabolic diseases increased substantially in the first decade of this century [8] with little change since, despite national initiatives to address the problem [9].

South Africa has the largest population living with HIV/AIDS world-wide [9] and the largest ART programme, with nearly 2 million people on treatment by 2011 [9]. The scale-up of antiretroviral therapy and declining mortality due to improved survival of people living with HIV/AIDS suggests an increased burden of cardiometabolic diseases and associated risk factors [10]. The growing need for chronic care of both HIV/AIDS and cardiometabolic diseases, in South Africa and regionally, highlights the importance of greater understanding of the burden of cardiometabolic diseases in this population [11].

In the present study, based on cross-sectional data from rural South Africa, we aimed to examine the factors associated with higher cardiometabolic risk, and to describe the association of cardiometabolic risk factors with HIV-status.

## Methods

### Setting and participants

We conducted a cross-sectional HIV/cardiometabolic risk factor survey in 2010 – 2011 using an age-sex stratified random sample of those aged 15+ in the Agincourt sub-district, situated in rural north-eastern South Africa adjacent to the country’s border with Mozambique. The sub-district has been under health and demographic surveillance since 1992, with collection of longitudinal data on all vital events and possible social, economic and environmental modifiers [12,13]. It is one of South Africa’s poorest areas, characterised by limited subsistence farming, shortage of services and high levels of migrant labor coupled with limited local employment opportunities. The area characteristics and the surveillance system are explained in more detail elsewhere [14].

For this cross-sectional study we randomly selected 7662 individuals ages 15+, stratified by age and sex, from an eligible population of 34,413 using the 2009 census update as the sampling frame [15]. In addition, we included 284 adults over age 50 years who participated in the INDEPTH-WHO/SAGE (http://www.who.int/healthinfo/sage/en/) ageing and adult health study in 2006 [16]. Of these individuals, 4362 consented to be interviewed and tested; we restricted our estimation sample to ages 18+ with complete covariate data (n = 3641). We used an indicator of household socioeconomic status [17] from the 2009 census update.

### Ethical approval

The study received ethical approvals from the University of the Witwatersrand Human Research Ethics Committee and the Mpumalanga Provincial Research and Ethics Committee. Written consent to participate (or parental consent and assent for minors ages 15–17) was obtained for all participants.

### Availability of supporting data

Data extraction of the Agincourt HDSS can be requested from Dr. F. Xavier Gómez-Olivé (F.Gomez-OliveCasas@wits.ac.za). Complete data sharing and collaboration details are available elsewhere [14].

### Procedures

A questionnaire on cardiometabolic risk factors, medication use and sexual behaviour was administered by experienced local fieldworkers who visited households up to three times to recruit the sample population. Five dried blood spots provided measures of HIV-status (HIV-, untreated HIV+, and treated HIV+). Treatment status was assessed by self-report. Point-of-care instruments were used to measure glycaemia, total cholesterol and lipid sub-fractions. Respondents were asked about smoking and alcohol history, physical activity, dietary intake, time since the last meal and whether they were using antiretroviral therapy. Physical measurements included height, weight and waist circumference using a flexible stadiometer (Seca, Hamburg, Denmark); and Analysis Scale Body Check (Seca, Hamburg, Denmark). Blood pressure was measured three times with a Boso blood pressure instrument (BOSCH + SOHN, Jungingen, Germany) three minutes apart, taken on the left wrist with participant in sitting positions and at rest; taking the average of the last two measurements. Random blood glucose was measured with a Caresens POP blood glucose meter (i-Sens, Nowon-gu, Seoul Korea); and total cholesterol and lipid sub-fractions were measured using a Cardiochek instrument (Polymer Technology Systems Inc., Indianapolis, IN USA). Socio-demographic characteristics such as years of formal education and employment were extracted from the existing surveillance system database.

### Variables used

To evaluate the association between cardiometabolic risk factors and HIV status, we determined different cut-offs for nine indicators by referring to published literature and aimed, whenever possible, to select an internationally recognized definition: high waist circumference as greater than 102 cm for men and 88 cm for women [18]; hypertension as systolic blood pressure greater than or equal to 140 mmHg or diastolic blood pressure greater than or equal to 90 mmHg or current use of antihypertensive medication [18]; high LDL cholesterol as greater than 3 mmol/l [19]; low HDL cholesterol as less than 1.03 mmol/l for men and 1.29 mmol/l for women [18]; high triglycerides as greater than or equal to 1.7 mmol/l [18]; high total cholesterol/ HDL ratio as greater than 8 [19]; diabetes as blood glucose (fasting or not; most of the blood samples were non-fasting) greater than or equal to 11.1 mmol/L [19]; obesity as body mass index of (BMI; kg/m^2^) 30 or higher [18]; and HIV-serostatus (HIV-, untreated HIV+, and treated HIV+). Personal and household socio-demographic characteristics included sex, age, formal education received (none, 1–5, 6+ years), smoking history (ever smoked or not), physical activity (low, moderate, high) [20], household asset score (in tertiles), employment status (currently employed or not), and alcohol frequency in the past month (no use, 1–3 days/month, 1–4 days/week, or 5+ days/week).

### Statistical methods

We calculated the unadjusted prevalence of HIV and cardio-metabolic risk factors by sex; and age-adjusted prevalence using the Agincourt 2009 census population. We used logistic regression to assess associations between cardiometabolic risk factors and HIV-status and a number of socio-demographic variables including sex, age, formal education, smoking history, physical activity, household asset score, employment status, and alcohol intake in the past month. We first fitted baseline models including HIV-status and covariates assumed to be unaffected by HIV-status (education and smoking history). We then estimated the full model with all covariates to determine if these factors moderated the association of HIV-status with each outcome. The HIV-status coefficients were not substantively changed by including all covariates so we present the full estimation results. All analyses were completed using STATA 12.1 and included sampling weights [21].

## Results

Demographic and lifestyle variables are presented in Table 1. Nearly two-thirds of the study sample were women, reflecting the demographic effect of widespread male-oriented migrant labour patterns in the study setting. Very few women reported ever smoking or drinking alcohol in the past month.

**Table 1:** **Demographic and lifestyle variables by sex, Agincourt sub-district, South Africa, 2010–2011 [All estimates include sampling weights; percentages may not sum to 100 due to rounding]**

	**Women (%) (N = 2163)**	**Men (%) (N = 1478)**
Age (years)		
18-29	38	64
30-49	31	15
50+	31	21
Formal education, years		
None	23	11
1-5	10	10
6+	67	80
Ever smoked	1	21
Currently employed	14	15
Alcohol frequency, past 30 days		
None	94	67
1 – 3 days/month	4	17
1 – 4 days/week	2	12
5+ days/week	1	4
Physical activity score^a^		
Low	47	38
Moderate	34	40
High	18	22

^a^Based on the International Physical Activity Questionnaire (IPAQ).

Age-adjusted adult HIV prevalence was 26% for women (95% CI 24–28) and 19% for men (95% CI 17–21) (Table 2). Overall measures of body shape and composition (waist circumference and BMI) showed markedly higher levels of abdominal and general obesity in women. There was no difference in the age-adjusted prevalence of hypertension by sex. Prevalence of hypertension was similar for the whole population (including HIV + and HIV -), and for those HIV negative only.

**Table 2:** **Unadjusted and age-adjusted prevalence of HIV and cardiometabolic risk factors by sex, Agincourt sub-district, South Africa, 2010–2011 [All estimates include sampling weights; age adjustment based on 2009 Agincourt census population; figures are % (with 95% confidence intervals]**

	**Women**		**Men**		
	**Unadjusted**	**Age-adjusted**	**Unadjusted**	**Age-adjusted**	**p-value (age-adjusted)**
HIV+	23	26	10	19	<0.001
	[21, 24]	[24, 28]	[9, 11]	[17, 21]	
Hypertension^a^ (whole sample)	40	39	30	37	0.301
	[38, 43]	[37, 41]	[27, 33]	[35, 40]	
Hypertension^a^ (HIV negative)	40	40	29	37	0.233
	[38, 43]	[37, 42]	[26, 32]	[35, 41]	
High waist circumference^b^	42	43	4	6	<0.001
	[40, 45]	[41, 45]	[3, 5]	[5, 8]	
Probable diabetes^c^	3	2	1	2	0.328
	[2, 4]	[2, 3]	[1, 2]	[1, 3]	
Obesity^d^	25	26	5	7	<0.001
	[23, 27]	[24, 28]	[3, 6]	[5, 8]	
High triglycerides^e^	23	22	20	24	0.096
	[21, 25]	[20, 23]	[17, 22]	[22, 27]	
High LDL cholesterol^f^	31	28	14	17	<0.001
	[28, 33]	[25, 30]	[11, 16]	[15, 20]	
Low HDL cholesterol^g^	28	29	16	12	<0.001
	[26, 30]	[27, 32]	[14, 19]	[10, 14]	
Any condition requiring chronic care^h^	54	56	36	49	<0.001
	[52, 57]	[53, 58]	[33, 40]	[46, 51]	

^a^Systolic blood pressure greater than or equal to 140 mmHg, or diastolic blood pressure greater than or equal to 90 mmHg or use of antihypertensive medication.

^b^Greater than 102 cm for men and 88 cm for women.

^c^Random blood glucose greater than or equal to 11.1.

^d^Body mass index (kg/m^2^) 30 or higher.

^e^Greater than or equal to 1.7 mmol/l.

^f^Greater than or equal to 3.0 mmol/l.

^g^Less than 1.04 mmol/l for men and 1.29 mmol/l for women.

^h^Either HIV+, hypertensive or diabetic.

Median values of cardiometabolic risk factors by sex, age-group and HIV and ART treatment status are shown in (Additional file 1: Table S1). Prevalence of cardiometabolic risk factors by sex, age-group, HIV and ART treatment status are shown in (Additional file 1: Figure S1).

Results of the logistic regression are shown in Tables 3 and 4. We excluded the total cholesterol/HDL ratio from the logistic regression since there were only eight individuals with a value greater than 8.

**Table 3:** **Influence of HIV serostatus, treatment status, and personal and household attributes on cardiometabolic risk among women in Agincourt sub-district 2010–2011 [All estimates include sampling weights]**

	**Odds ratio [95% CI]**						
	**Obesity** ^**c**^	**High waist circumference** ^**d**^	**Diabetes** ^**e**^	**Hypertension** ^**f**^	**High triglycerides** ^**g**^	**High LDL cholesterol** ^**h**^	**Low HDL cholesterol** ^**i**^
HIV status							
HIV-	−	−	−	−			
HIV+, no treatment	0.64	0.67	0.4	0.82	1.19	0.53	1.38
	[0.49, 0.85]	[0.52, 0.86]	[0.15, 1.04]	[0.63, 1.07]	[0.90, 1.57]	[0.38, 0.73]	[1.06, 1.81]
HIV+, on treatment	0.27	0.5	0.64	0.74	1.65	0.83	0.56
	[0.15, 0.46]	[0.33, 0.76]	[0.18, 2.23]	[0.49, 1.10]	[1.10, 2.48]	[0.51, 1.33]	[0.33, 0.95]
Age groups							
18-29	0.27	0.21	0.22	0.29	0.51	0.58	2.08
	[0.20, 0.37]	[0.16, 0.28]	[0.07, 0.76]	[0.22, 0.39]	[0.37, 0.71]	[0.40, 0.83]	[1.60, 2.72]
30-49	−	−	−	−			
50+	1.03	1.73	4.53	2.39	1.57	2.79	0.55
	[0.78, 1.35]	[1.33, 2.24]	[2.37, 8.64]	[1.83, 3.10]	[1.18, 2.08]	[2.09, 3.72]	[0.40, 0.76]
SES tertiles^a^							
1st (lowest)	−	−	−	−			
2nd	1.65	1.46	1.36	1.1	1.3	1.04	0.9
	[1.26, 2.15]	[1.15, 1.87]	[0.71, 2.61]	[0.86, 1.42]	[0.98, 1.71]	[0.77, 1.40]	[0.68, 1.19]
3rd (highest)	1.92	1.84	1.72	1.03	1.35	1.09	0.84
	[1.47, 2.52]	[1.43, 2.37]	[0.94, 3.16]	[0.79, 1.33]	[1.02, 1.79]	[0.81, 1.48]	[0.62, 1.13]
Currently employed	1.28	1.41	1	1.21	1.12	0.84	0.89
	[0.99, 1.67]	[1.08, 1.84]	[0.46, 2.17]	[0.93, 1.57]	[0.84, 1.49]	[0.61, 1.15]	[0.66, 1.20]
Ever smoked	0.41	0.5	1.18	2.2	1.33	0.76	1.25
	[0.16, 1.09]	[0.23, 1.06]	[0.25, 5.43]	[0.86, 5.60]	[0.59, 3.01]	[0.34, 1.70]	[0.46, 3.41]
Physical activity^b^							
Low	−	−	−	−			
Moderate	0.93	1.04	1.22	0.72	1.06	0.77	0.96
	[0.74, 1.17]	[0.83, 1.31]	[0.70, 2.14]	[0.57, 0.90]	[0.83, 1.36]	[0.59, 1.01]	[0.74, 1.24]
High	0.85	0.88	0.94	0.71	0.91	0.77	0.84
	[0.62, 1.17]	[0.66, 1.17]	[0.43, 2.06]	[0.53, 0.96]	[0.65, 1.27]	[0.54, 1.10]	[0.60, 1.18]
Alcohol frequency, past month							
Not in past 30 days	−	−	−	−			
1 – 3 days/month	0.54	1.14	*Omitted*	1.08	1.32	0.6	0.77
	[0.27, 1.09]	[0.64, 2.05]		[0.63, 1.88]	[0.68, 2.56]	[0.28, 1.26]	[0.38, 1.58]
1 – 4 days/week	0.89	1.11	0.69	0.54	1.6	0.49	0.65
	[0.38, 2.05]	[0.56, 2.19]	[0.15, 3.17]	[0.29, 0.97]	[0.69, 3.71]	[0.22, 1.06]	[0.22, 1.97]
5+ days/week	0.14	0.72	*Omitted*	0.75	1.91	1.05	0.36
	[0.02, 1.05]	[0.23, 2.24]		[0.24, 2.32]	[0.35, 10.32]	[0.27, 4.06]	[0.03, 3.88]
Formal education							
None	−	−	−	−			
1 – 5 years	1.45	1.3	0.9	0.74	0.87	1.17	1.18
	[1.04, 2.01]	[0.94, 1.80]	[0.43, 1.91]	[0.53, 1.03]	[0.61, 1.23]	[0.82, 1.67]	[0.77, 1.79]
6+ years	1.45	1.38	1.46	0.88	0.93	0.93	1.36
	[1.08, 1.95]	[1.04, 1.84]	[0.78, 2.71]	[0.66, 1.17]	[0.68, 1.25]	[0.68, 1.27]	[0.97, 1.92]
N	2057	2064	1984	2064	2064	1601	2064

^a^Based on an household asset index score in 2009.

^b^Based on the International Physical Activity Questionnaire (IPAQ).

^c^Body mass index (kg/m^2^) 30 or higher.

^d^Greater than 88 cm.

^e^Blood glucose greater than or equal to 11.1.

^f^Systolic blood pressure greater than or equal to 140 mmHg, or diastolic blood pressure greater than or equal to 90 mmHg or use of antihypertensive medication.

^g^Greater than or equal to 1.7 mmol/l.

^h^Greater than or equal to 3.0 mmol/l.

^i^Less than 1.29 mmol/l.

**Table 4:** **Influence of HIV serostatus, treatment status, and personal and household attributes on cardiometabolic risk among men in Agincourt sub-district 2010–2011 [All estimates include sampling weights]**

	**Odds ratio [95% CI]**						
	**Obesity** ^**c**^	**High waist circumference** ^**d**^	**Diabetes** ^**e**^	**Hypertension** ^**f**^	**High triglycerides** ^**g**^	**High LDL cholesterol** ^**h**^	**Low HDL cholesterol** ^**i**^
HIV status							
HIV-	−	−	−	−			
HIV+, no treatment	0.78	0.42	1.13	0.76	0.81	0.42	1.15
	[0.44, 1.40]	[0.22, 0.82]	[0.46, 2.75]	[0.52, 1.11]	[0.55, 1.18]	[0.25, 0.72]	[0.66, 2.01]
HIV+, on treatment	*Omitted* ^*j*^	*Omitted* ^*j*^	1	1.23	1.4	0.57	0.45
			[0.20, 5.11]	[0.70, 2.17]	[0.79, 2.50]	[0.27, 1.18]	[0.13, 1.56]
Age groups							
18-29	0.22	0.15	*Omitted* ^*j*^	0.48	0.37	0.31	1.74
	[0.10, 0.50]	[0.06, 0.39]		[0.33, 0.68]	[0.25, 0.54]	[0.18, 0.53]	[1.07, 2.83]
30-49	−	−	−	−			
50+	0.96	1.59	4.15	2.59	1.21	2.04	0.3
	[0.49, 1.90]	[0.88, 2.86]	[1.82, 9.50]	[1.79, 3.76]	[0.80, 1.83]	[1.27, 3.29]	[0.13, 0.71]
SES tertiles^a^							
1st (lowest)	−	−	−	−			
2nd	1.06	1.31	1.34	1.57	1.06	0.86	1.22
	[0.43, 2.61]	[0.59, 2.90]	[0.51, 3.51]	[1.06, 2.33]	[0.69, 1.63]	[0.50, 1.47]	[0.70, 2.11]
3rd (highest)	2.13	2.98	2.29	1.44	1.83	0.78	1.02
	[0.97, 4.68]	[1.48, 6.00]	[0.93, 5.62]	[0.98, 2.11]	[1.20, 2.79]	[0.46, 1.32]	[0.57, 1.82]
Currently employed	1.34	1.18	0.84	1.47	1.72	1.14	0.8
	[0.87, 2.07]	[0.75, 1.87]	[0.41, 1.73]	[1.04, 2.09]	[1.21, 2.43]	[0.76, 1.70]	[0.48, 1.32]
Ever smoked	0.7	0.88	1.28	0.84	0.76	0.97	1.12
	[0.29, 1.66]	[0.39, 2.00]	[0.59, 2.80]	[0.60, 1.17]	[0.55, 1.06]	[0.58, 1.63]	[0.63, 1.99]
Physical activity^b^							
Low	−	−	−	−			
Moderate	1.91	1.59	0.85	0.84	1.01	0.76	1.06
	[1.12, 3.28]	[0.94, 2.69]	[0.40, 1.82]	[0.59, 1.18]	[0.69, 1.48]	[0.49, 1.18]	[0.64, 1.75]
High	0.42	0.45	2.8	1.02	1.6	0.61	0.89
	[0.17, 1.02]	[0.12, 1.63]	[0.94, 8.36]	[0.62, 1.67]	[0.93, 2.74]	[0.26, 1.44]	[0.46, 1.71]
Alcohol frequency, past month							
Not in past 30 days	−	−	−	−			
1 – 3 days/month	0.8	0.99	0.54	1.97	1.07	0.44	0.99
	[0.33, 1.95]	[0.41, 2.40]	[0.19, 1.57]	[1.29, 3.00]	[0.67, 1.71]	[0.26, 0.75]	[0.57, 1.72]
1 – 4 days/week	0.49	0.38	0.6	1.18	2.21	1.31	0.26
	[0.22, 1.09]	[0.16, 0.92]	[0.18, 1.96]	[0.77, 1.83]	[1.37, 3.55]	[0.66, 2.57]	[0.10, 0.65]
5+ days/week	0.84	0.42	0.22	1.05	1.13	0.16	0.16
	[0.24, 2.95]	[0.15, 1.20]	[0.03, 1.97]	[0.61, 1.81]	[0.61, 2.08]	[0.06, 0.44]	[0.02, 1.22]
Formal education							
None	−	−	−	−			
1 – 5 years	2.31	1.66	0.62	0.81	1.04	1.34	0.47
	[0.91, 5.87]	[0.85, 3.24]	[0.22, 1.70]	[0.51, 1.29]	[0.60, 1.80]	[0.77, 2.33]	[0.16, 1.38]
6+ years	1.37	1.54	1.47	0.76	1.29	1.48	0.37
	[0.60, 3.11]	[0.80, 2.98]	[0.65, 3.35]	[0.49, 1.17]	[0.79, 2.11]	[0.82, 2.65]	[0.13, 1.01]
N	1293	1300	940	1369	1369	1031	1369

^a^Based on an household asset index score in 2009.

^b^Based on the International Physical Activity Questionnaire (IPAQ).

^c^Body mass index (kg/m^2^) 30 or higher.

^d^Greater than 102 cm.

^e^Blood glucose greater than or equal to 11.1.

^f^Systolic blood pressure greater than or equal to 140 mmHg, or diastolic blood pressure greater than or equal to 90 mmHg or use of antihypertensive medication.

^g^Greater than or equal to 1.7 mmol/l.

^h^Greater than or equal to 3.0 mmol/l.

^i^Less than 1.04 mmol/l.

^j^Category omitted due to small sample size.

For women, after adjusting for age, education, household SES and employment status, as well as smoking status, physical activity, and alcohol use, untreated HIV+ status was associated with lower odds of obesity (OR 0.64; 95% CI 0.49-0.85), high waist circumference (OR 0.67; 95% CI 0.52-0.86), and high LDL (OR 0.53; 95% CI 0.38-0.73) relative to HIV- female respondents; and higher odds of low HDL (OR 1.38; 95% CI 1.06-1.81) (Table 3). Treated HIV+ women were at lower odds of obesity (OR 0.27; 95% CI 0.15-0.46), high waist circumference (OR 0.50; 95% CI 0.33-0.76), and low HDL (OR 0.56; 95% CI 0.33-0.95) relative to HIV- women. There was an increased odds of high triglycerides (OR 1.65; 95% CI 1.10-2.48) among treated women compared with HIV- women.

The odds of elevated risk markers in women increased with age for all cardiometabolic risk factors except low HDL cholesterol (in which the odds decreased with age). Living in a higher SES household relative to the lowest SES households (first quintile) increased the odds of obesity, high waist circumference and high triglycerides. Current employment was associated with a higher waist circumference; and having any formal education increased the odds of obesity. Being physically active was associated with decreased odds of hypertension.

For men, after adjusting for age, education, household SES and employment status, as well as smoking status, physical activity and alcohol use, untreated HIV+ status was associated with reduced odds of high waist circumference (OR 0.42; 95% CI 0.22-0.82) and raised LDL cholesterol (OR 0.42; 95% CI 0.25-0.72) relative to HIV- male respondents (Table 4). Age associations with cardiometabolic risk factors were comparable to those of women but the association was only statistically significant for hypertension, high LDL cholesterol and low HDL cholesterol. Being in the highest SES household relative to the lowest increased the odds of high waist circumference and raised triglycerides. Risk of hypertension increased with increasing SES, although the odds ratio for hypertension in the highest level of SES relative to the lowest did not quite reach statistical significance. Current employment increased the odds of hypertension and high triglycerides. Trends with alcohol consumption were inconsistent.

## Discussion

We have described the prevalence of HIV and cardiometabolic risk factors in a rural South African population 18 years and older. We found a high prevalence of HIV and cardiovascular risk factors, particularly hypertension, in this population.

A strength of the study is that the sample was drawn from a well-established health and demographic surveillance system with clear field and data entry procedures to ensure the quality of information [14,22]. We were thus able to include socio-demographic variables for participants and could calculate age-standardised prevalence estimates.

We assessed ART use by self-report which was likely underestimated since there remains considerable stigma associated with HIV [23,24]. Moreover, we acknowledge that some forms of ART used by our respondents may affect their lipid levels and insulin resistance [25,26], and we cannot isolate this possible effect. We were not able to measure fasting glucose because of the distances between households and road conditions, which meant that early morning visits to participants were not practical. Also resulting from logistical considerations, metabolic risk factors were measured using point of care instruments instead of laboratory analysis. We used internationally recognized cut-offs for all cardiometabolic risk factors but it is possible that using different cut-offs results may have varied.

The WHO-SAGE study of older people conducted in 2006 and covering six countries in Asia, Africa and Latin America found that South Africa had the highest recorded levels of hypertension (78%) [27]. This was higher than findings from a similar study in the Agincourt sub-district where the prevalence of hypertension in the population 50 years and above was 57% [16]. In the present study, which includes all age groups from 18 years, the age-standardised prevalence was 39% in women and 37% in men. It is clear that South Africa is facing an epidemic of hypertension.

A recent systematic review considered the association of HIV and ART with cardiometabolic risk factors in sub-Saharan Africa [28]. Using data from 52 studies, which reported on nearly 30,000 individuals, the review found that untreated HIV was associated with lower BMI and HDL and raised triglycerides. These findings were confirmed in this study for women. The review also reported lower systolic and diastolic blood pressure in people with HIV but we did not find any significantly different risk in recorded hypertension. Our results and the results of the review highlight the importance of establishing the relationship between HIV status, use of ART and cardiometabolic diseases. In the region with the largest number of people infected with HIV and an increasing number of people suffering from cardiovascular diseases, establishing the association between them is an urgent priority.

The findings on cardiovascular risk factors in people taking ART could not be established in our study as we were limited to self-reported ART use. Our finding of lower BMI in those on ART may be explained by the fact that when data were collected, national policy was that ART should be initiated only when the CD4 count was below 200 cells/mm^3^ [29] – meaning that these people were already very sick when treatment was initiated.

## Conclusions

Sub-Saharan Africa is facing parallel and interacting epidemics of HIV and cardiometabolic disease risk that are fuelled by complex health and social transitions. Our results indicate both a high prevalence of HIV and of hypertension in a poor, rural South African population. Although we did not find any interaction between these two conditions, it is inevitable that some people will be affected by both and our results show that around half of the adult population (56% of women and 49% of men, Table 2) are in need of long-term care for at least one of HIV, hypertension or diabetes. This carries serious implications for the South African health system and strengthens our prior argument, based on comprehensive cause-of-death trends, that delivery of functionally integrated chronic care will be critical to meeting health needs at the primary care level [7].

We have also confirmed the results of an earlier systematic review, that the presence of HIV has an adverse effect on HDL cholesterol and triglycerides, suggesting that people living with HIV may be at increased risk of cardiovascular disease, and that there may be an acceleration of the already increasing prevalence of cardiometabolic conditions. As yet, we have only measured self-reported ART status. However, in the context of continued ART rollout, an ageing population, and increasing HIV prevalence in the older population, future monitoring of ART access and use, and the interaction with cardiometabolic risk factors is urgently needed to understand and quantify the challenges both to the health service and the wider society.

## Additional file

Additional file 1: Table S1. (median values of cardiometabolic risk factors) and **Figure S1.** (prevalence of cardiometabolic risk factors).
