# Supplementary material for: Cardiometabolic disease risk and HIV status in rural South Africa: establishing a baseline
Source: BMC Public Health. 2015 Feb 12;15:135. doi: 10.1186/s12889-015-1467-1 (PMC4335669; doi:10.1186/s12889-015-1467-1)

Table S1. Median values of cardiometabolic risk factors, by sex, age group, and HIV sero- and treatment status, Agincourt sub-district, South Africa, 2010-2011
[all estimates include sampling weights]

	Women											
	18-29 (N = 516)				30-49 (N = 861)				50+ (N = 699)			
	Total	HIV-	HIV+, no treatment	HIV+, on treatment	Total	HIV-	HIV+, no treatment	HIV+, on treatment	Total	HIV-	HIV+, no treatment	HIV+, on treatment
Body mass index	23·54	23·26	24·62	24·36	27·4	28·73	25·72	25·5	26·87	27·06	25·91	23·63
Waist circumference	78	77·3	82	85·6	90·2	93·2	85·5	86	94·2	95·6	89	85·5
Blood glucose	5·1	5·1	5·1	4·8	5·2	5·3	5·1	5·3	5·7	5·8	5·4	5·3
Systolic BP	117·5	118·5	115·5	112·5	125·5	128	123	119	136·5	137	130·5	126
Diastolic BP	78	78·5	76·5	79·5	86·5	87·5	86	82	88	88	88	85·5
Total cholest/HDL	2·8	2·81	2·79	2·72	2·84	2·88	2·74	2·74	3·17	3·2	2·98	2·93
Triglycerides	0·77	0·73	0·84	0·74	1·04	1·03	1·01	1·18	1·3	1·29	1·34	2·25
LDL cholesterol	1·88	1·95	1·72	1·6	2·32	2·39	2·08	2·35	3·07	3·19	2·25	2·89
HDL cholesterol	1·4	1·42	1·27	1·59	1·58	1·59	1·58	1·64	1·77	1·76	1·72	2·23
	**Men**											
	18-29 (N = 429)				30-49 (N = 456)				50+ (N = 503)			
	Total	HIV-	HIV+, no treatment	HIV+, on treatment	Total	HIV-	HIV+, no treatment	HIV+, on treatment	Total	HIV-	HIV+, no treatment	HIV+, on treatment
Body mass index	20·99	20·98	21·55	25·87	22·96	23·49	22·32	20·88	23·41	23·69	22·03	21·68
Waist circumference	74·6	74·5	74	89·2	82·3	84	80·2	79·2	87	88·1	83·3	86
Blood glucose	4·9	4·9	4·7	6	5·1	5·1	5·1	5·3	5·6	5·7	5·3	5·6
Systolic BP	124	124·5	123·75	121	127	127	127·5	125·5	136	138	125·5	127
Diastolic BP	78·5	78·5	80·5	81·5	85	85	85·5	89	87·5	88·5	83·5	81
Total cholest/HDL	2·56	2·57	2·37	2·27	2·8	2·96	2·54	2·75	2·78	2·77	2·83	2·74
Triglycerides	0·83	0·82	0·92	1·48	1·19	1·18	1·18	1·3	1·18	1·15	1·19	1·36
LDL cholest	1·69	1·69	1·66	1·55	2·03	2·23	1·8	2·24	2·42	2·55	2·29	2·41
HDL cholest	1·32	1·32	1·48	1·75	1·52	1·52	1·48	1·7	1·68	1·69	1·48	1·74

Figure S1. Prevalence of cardiometabolic risk factors, by sex, age group, and HIV sero- and treatment status, Agincourt sub-district, South Africa, 2010-2011
[all estimates include sampling weights]

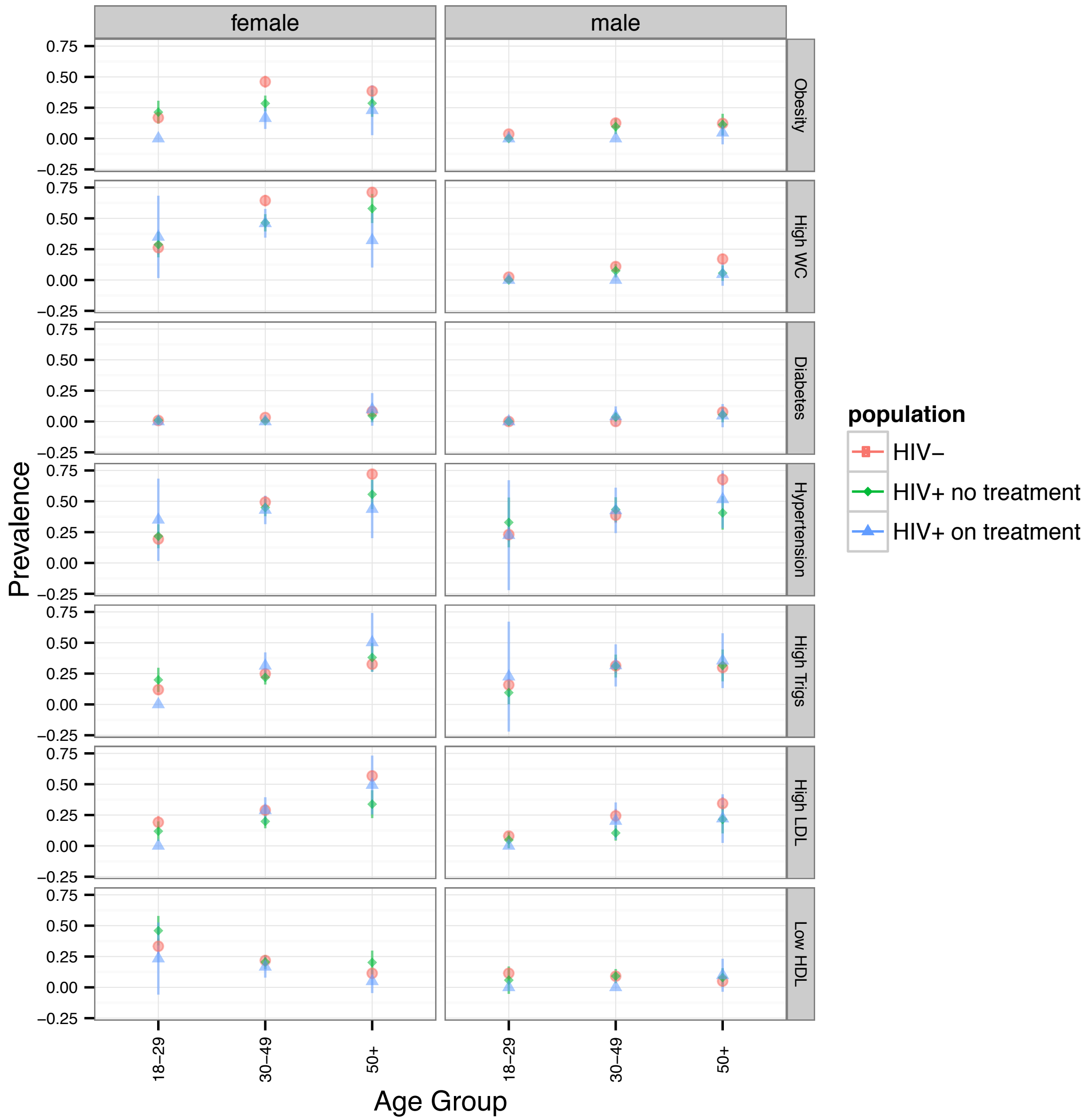

Supplement: Additional file 1: Table S1. — (median values of cardiometabolic risk factors) and Figure S1. (prevalence of cardiometabolic risk factors). [file 12889_2015_1467_MOESM1_ESM.pdf]
